# Rapid conversion of replicating and integrating *Saccharomyces cerevisiae* plasmid vectors via Cre recombinase

**DOI:** 10.1093/g3journal/jkab336

**Published:** 2021-10-02

**Authors:** Daniel P Nickerson, Monique A Quinn, Joshua M Milnes

**Affiliations:** 1 Department of Biology, California State University, San Bernardino, San Bernardino, CA 92407, USA; 2 Department of Biochemistry, University of Washington School of Medicine, Seattle, WA 98195-3750, USA

**Keywords:** Cre recombinase, homologous recombination, plasmid, shuttle vector, replication, molecular cloning, genetic engineering, yeast

## Abstract

Plasmid shuttle vectors capable of replication in both *Saccharomyces cerevisiae* and *Escherichia coli* and optimized for controlled modification *in vitro* and *in vivo* are a key resource supporting yeast as a premier system for genetics research and synthetic biology. We have engineered a series of yeast shuttle vectors optimized for efficient insertion, removal, and substitution of plasmid yeast replication loci, allowing generation of a complete set of integrating, low copy and high copy plasmids via predictable operations as an alternative to traditional subcloning. We demonstrate the utility of this system through modification of replication loci via Cre recombinase, both *in vitro and in vivo*, and restriction endonuclease treatments.

## Introduction

Yeast (*Saccharomyces cerevisiae*) has long served as a premier experimental system for exploring eukaryotic genetics and cell biology (Botstein and Fink 2011) and has more recently emerged as a preferred system in synthetic biology (Lee *et al.* 2015). A key component of the yeast genetic toolkit is the availability of shuttle vectors, plasmids capable of replication and selection when introduced into either a yeast or *Escherichia coli* cell (Sikorski and Hieter 1989; Da Silva and Srikrishnan 2012). In their original design, yeast shuttle vectors would typically be modified *in vitro* and subsequently propagated in bacterial cells to generate the desired gene construct that could be later introduced into yeast to study eukaryotic cell biology. In current practice, the classical bacteria-then-yeast workflow is often turned on its head by gene synthesis techniques that capitalize upon the readiness of yeast to piece together compatible DNA fragments via homologous recombination (Gibson 2009), so initial gene synthesis and molecular cloning steps often start inside the yeast cell.

Yeast shuttle vectors vary in their selectable markers, typically genes for auxotrophic rescue or antibiotic resistance, and in the presence or absence of a yeast replication locus. Yeast integrating plasmids lack an independent replication locus and must be incorporated into a chromosome in order to replicate. Low copy, centromeric plasmids contain an autonomous replicating sequence (ARS) to initiate DNA replication and a centromere (*CEN*) to support plasmid partitioning during cell division. High copy, episomal vectors carry the 2 µ circle replicating origin, FRT recombinase site and *STB* partitioning locus (Rizvi *et al.* 2018). Low and high copy plasmids offer convenience for gene expression studies and functional analyses, but both varieties of replicating plasmids have demonstrated instability in their copy numbers (Futcher and Carbon 1986; Mead *et al.* 1986; Resnick *et al.* 1990). When considering the possible toxicity of plasmid-encoded gene or combination of genes, or even the energetic cost of maintaining the plasmid itself (Mead *et al.* 1986), unstable copy numbers of centromeric and 2 µ plasmids can present clear obstacles to experimental execution and interpretation. To ensure even copy number and consistent level of gene expression, some plasmid-encoded genes must be integrated into a chromosome, but such necessity is often realized after experimentation with expression from replicating plasmids. Ability to switch between low copy, high copy, and integrating shuttle vector strategies is a key feature of the yeast genetics toolkit.

Previous efforts in improving resources for yeast shuttle vectors have focused heavily on engineering sequences flanking the yeast selectable marker loci to offer convenient removal and remodeling (Gueldener *et al.* 2002; Carter and Delneri 2010; Fang *et al.* 2011; Chee and Haase 2012; Agaphonov and Alexandrov 2014; Jensen *et al.* 2014; Siddiqui *et al.* 2014), either in the plasmid or after chromosomal integration. We believe a similar level of utility is achieved by engineering the yeast replication loci of shuttle vectors to be removed and remodeled, but by comparison the replication loci of the shuttle vectors have been neglected. The example of which we are aware (Chou *et al.* 2015) engineered low copy yeast shuttle vectors (pXR series) to convert to integrating vectors via Cre recombinase, but the pXR series does not include high copy plasmids and does not include additional options to remove replication loci in targeted fashion via restriction endonucleases.

We have modified the popular family of pRS shuttle vectors (Sikorski and Hieter 1989; Christianson *et al.* 1992) plasmid shuttle vectors to include both low and high copy yeast replication loci flanked by LoxP sites or triplicate endonuclease cut sites. We demonstrate the utility and flexibility of this system through *in vivo* and *in vitro* remodeling of the yeast replication loci and the rapid generation of a complete suite of integrating, low copy and high copy plasmids to support functional analysis in yeast.

## Materials and methods

### Media and reagents

Standard methods were used for culture and storage of yeast and bacteria (Guthrie and Fink 2002). All media and reagents were purchased from Sigma-Aldrich (Saint Louis, MO, USA) or Thermo Fisher (Waltham, MA, USA), unless otherwise specified. All enzymes were purchased from New England Biolabs (Ipswich, MA, USA) unless otherwise specified. High fidelity KOD Hot Start polymerase was purchased from Novagen/EMD Millipore (Darmstadt, Germany). DNA restriction digests, T4 DNA ligase reactions, and PCR reactions were all performed according to manufacturer instructions. Oligonucleotide (primer) synthesis was performed by Integrated DNA Technologies (Corralville, IA, USA). Hyperladder I (Bioline) was used as linear DNA size standard.

### DNA manipulations and reagents

Strains and plasmids used in this study are described in Table 1. Oligonucleotides used in this study are described in Supplemental Materials (Supplementary Table S1). Because publicly available DNA sequences for pRS403, pRS404, pRS405, and pRS406 (Genbank accession numbers U03443.1, U03444.1, U03445.1, and U03451.1, respectively) contain inaccuracies (Chee and Haase 2012), we re-examined the entirety of these plasmid sequences via Sanger DNA sequencing (performed by Genewiz, South Plainfield, NJ) to generate revised plasmid sequences and maps (available in SnapGene .dna file format in Supplemental Materials).

**Table 1:** Plasmids and strains used in this study

Name	Genotype/description	Source/reference	Addgene id
*Plasmids*			
pDN613	Amp^R^*HIS3 LoxP::CEN/ARSH4::LoxP*	This study	175373
pDN614	Amp^R^*TRP1 LoxP::CEN/ARSH4::LoxP*	(Shideler *et al.* 2015)	175374
pDN615	Amp^R^*LEU2 LoxP::CEN/ARSH4::LoxP*	(Paulsel *et al.* 2013)	175375
pDN616	Amp^R^*URA3 LoxP::CEN/ARSH4::LoxP*	(Nickerson *et al.* 2012)	175376
pDN623	Amp^R^*HIS3 LoxP::2*µ*::LoxP*	This study	175377
pDN624	Amp^R^*TRP1 LoxP::2*µ*::LoxP*	This study	175378
pDN625	Amp^R^*LEU2 LoxP::2*µ*::LoxP*	This study	175379
pDN626	Amp^R^*URA3 LoxP::2*µ*::LoxP*	This study	175380
pDN513	Amp^R^*HIS3 3X::CEN/ARSH4::3X*	This study	175365
pDN514	Amp^R^*TRP1 3X::CEN/ARSH4::3X*	This study	175366
pDN515	Amp^R^*LEU2 3X::CEN/ARSH4::3X*	This study	175367
pDN516	Amp^R^*URA3 3X::CEN/ARSH4::3X*	This study	175368
pDN523	Amp^R^*HIS3 3X::2*µ*::3X*	This study	175369
pDN524	Amp^R^*TRP1 3X::2*µ*::3X*	(Lobingier *et al.* 2014)	175370
pDN525	Amp^R^*LEU2 3X::2*µ*::3X*	This study	175371
pDN526	Amp^R^*URA3 3X::2*µ*::3X*	(Nickerson *et al.* 2012)	175372
pJM1	Amp^R^*URA3 LoxP::CEN/ARSH4::LoxP PRC1pr::GFP-CPS1*	This study	—
pJM3	Amp^R^*URA3 LoxP PRC1pr::GFP-CPS1*	This study	—
pDN314	Amp^R^*URA3 3X::2*µ*::3X SEC17*	(Lobingier *et al.* 2014)	—
pDN366	Amp^R^*URA3 3X::CEN/ARSH4::3X sec17^L291A/L292A^*	This study	—
pDN369	Amp^R^*URA3 3X::2*µ*::3X sec17^L291A/L292A^*	(Schwartz *et al.* 2017)	—
pDN370	Amp^R^*URA3 sec17^L291A/L292A^*	This study	—
pRS403	Amp^R^*HIS3*	(Sikorski and Hieter 1989)	—
pRS404	Amp^R^*TRP1*	(Sikorski and Hieter 1989)	—
pRS405	Amp^R^*LEU2*	(Sikorski and Hieter 1989)	—
pRS406	Amp^R^*URA3*	(Sikorski and Hieter 1989)	—
pRS415	Amp^R^*LEU2 CEN/ARSH4*	(Sikorski and Hieter 1989)	—
pRS425	Amp^R^*LEU2* 2 µ	(Christianson *et al.* 1992)	—
pGO45	Amp^R^*URA3* 2 µ *PRC1pr::GFP-CPS1*	(Odorizzi *et al.* 1998)	—
*E. coli*			
TOP10F’	*[lacI^q^ Tn10(tet^R^)] mcrA Δ(mrr-hsdRMS-mcrBC)φ80lacZ ΔM15 ΔlacX74 deoR nupG recA1 araD139Δ (ara-leu)7697 galU galK rpsL(Str^R^) endA1 λ^−^*	Invitrogen	—
N2114Sm	F^−^*recA λ-cre rpsL*	(Seifert *et al.* 1986)	—
*S. cerevisiae*			
SEY6210	*MATα leu2-3,112 ura3-52 his3-200 trp1-901 lys2-801 suc2-9*	(Robinson *et al.* 1988)	—
MBY3	SEY6210 *vps4Δ::TRP1*	(Babst *et al.* 1997)	—
JMY1	SEY6210 *GFP-CPS1* (pJM3::*URA3*)	This study	—
JMY2	MBY3 *GFP-CPS1* (pJM3::*URA3*)	This study	—

In constructing the pDN51x or pDN52x plasmid series, PCR primer pairs (DN652p & DN653p or DN1016p & DN1017p) were designed to amplify either the *CEN/ARSH4* locus or the high copy 2 µ locus from pRS415 or pRS425 templates, respectively, while incorporating AatII, AvrII, and SphI restriction sites (“3X”) flanking the loci. To construct the pDN61x plasmid series, PCR primer pairs (DN648p & DN649p or MQ1p & MQ2p) were designed to amplify *CEN/ARSH4* or 2 µ loci from pRS415 or pRS425 templates, respectively, while incorporating parallel LoxP sequences flanking the loci. After successful high-fidelity PCR amplification, template plasmid DNA was degraded by treatment with DpnI restriction enzyme prior to precipitation of PCR product and resuspension in 0.1M LiOAc 2 mM Tris pH 7.9. The resulting *LoxP::CEN/ARSH4::LoxP*, *LoxP::2*µ*::LoxP, 3X::CEN/ARSH4::3X* and *3X::2*µ*::3X* PCR products were co-transformed into yeast (*S. cerevisiae*) with AatII-digested pRS403, pRS404, pRS405 or pRS406 linearized vectors using a lithium acetate-based protocol described below. Homologous recombination of the replication loci and linearized integrating vectors yielded new, low- and high-copy replicating vectors. pDN5xx- and pDN6xx-series plasmid candidates were screened and confirmed by restriction digest, DNA sequencing, competence for Cre-mediated recombination, and ability to support yeast and bacterial colony growth after transformation and plating onto selective media.

Yeast high-efficiency DNA transformation protocol for recircularization of linearized plasmid vectors by homologous recombination with compatible DNA inserts was adapted from (Gietz *et al.* 1992). Cells were shaken overnight in YPD media at 30°C. Saturated cultures were diluted to OD_600_ ∼0.1 and shaken under identical conditions until cells reached log phase density (OD_600_ = 0.4–0.6). Cells were collected by low-speed centrifugation and rinsed in 0.1M LiOAc 2 mM Tris pH 7.9. Cell pellets were resuspended in 50 µl 0.1M LiOAc 2 mM Tris pH 7.9 containing either resuspended PCR product ('insert') or no PCR product as a negative control. Cell suspensions were further supplemented with 10–50 µg boiled salmon sperm DNA and approximately 25 ng linearized plasmid vector before dilution with 700 µl 40% PEG (w/v) in 0.1M LiOAc 2 mM Tris pH 7.9. Cell suspensions were vortexed 10–20 s and incubated at 30°C for 15–30 min. Cell suspensions were supplemented with 5% (v/v) DMSO and vortexed another 10 s prior to a heat shock incubation at 42°C for 30 min. Cells were collected by low-speed centrifugation and resuspended in 0.1M LiOAc 2 mM Tris pH 7.9 prior to spreading on selective agar media. The yeast transformation protocol is robust; variations in culture density, incubation times, and salmon sperm DNA concentration within the ranges described above do not produce qualitatively different results.

Plasmid DNA was recovered from yeast by DNA extraction using the “smash and grab” protocol (Rose *et al.* 1990) of glass bead cell lysis, phenol-chloroform extraction, and ethanol precipitation of the aqueous phase to yield genomic and plasmid DNA. Plasmids were separated from genomic DNA by electroporation of plasmids into TOP10F’ *E. coli* and selection of transformed cells on LB agar media with ampicillin (100 µg/ml). Plasmids were recovered from bacteria using a QIAgen plasmid miniprep kit (Qiagen, Valencia, CA, USA). Sanger DNA sequencing reactions of replication loci using flanking primers DN661p or DN837p were performed by Genewiz. Sequence alignments were performed using SnapGene software (GSL Biotech, San Diego, CA, USA).

All restriction endonuclease digests of plasmids were performed according to manufacturer’s instructions (New England Biolabs).

Cre-mediated removal of *LoxP-*flanked replication loci from pDN61x- and pDN62x-series vectors was accomplished *in vivo* by chemical transformation of an *E. coli* strain expressing Cre recombinase, N2114Sm (Seifert *et al.* 1986). Ampicillin-selected colonies were picked and grown in LB media supplemented with ampicillin (50 µg/ml) before plasmid extraction via QIAgen plasmid miniprep. Plasmid yields from N2114Sm host strain are low, so plasmid candidates purified from N2114Sm bacteria were subsequently ‘passaged’ in TOP10F’ *E. coli* (Invitrogen, Carlsbad, CA, USA) via chemical transformation and QIAgen plasmid miniprep extraction to obtain higher plasmid yields for analysis. All references in this study to plasmid “passaging” indicate plasmid transformation of *E. coli* and subsequent re-purification of the plasmid from the resulting transformed bacteria.

Cre-mediated removal of *LoxP-*flanked replication loci from pDN62x-series vectors was accomplished *in vitro* treating 250 ng plasmid with purified Cre recombinase enzyme (New England Biolabs) in 50 µl reaction per manufacturer instructions, incubating at 37°C for 30 min. Resulting polyclonal Cre recombinase reactions were heat inactivated and plasmid DNA was precipitated twice using ice cold 70% (v/v) ethanol prior to resuspension in 10 mM Tris pH 8, followed by restriction digest and electrophoretic analysis. For isolation of individual plasmid candidates resulting from *in vitro* Cre-treatment, the Cre-treated polyclonal pool was transformed into *E. coli* to yield ampicillin-selected colonies from which monoclonal plasmid candidate samples were purified for analysis. Oligonucleotide DN837p was used for Sanger DNA sequencing to confirm loss of replication loci in Cre-treated plasmids.

Plasmid pJM1 was constructed via PCR amplification of the *PRC1* promoter-driven *GFP-CPS1* cassette from plasmid template pGO45 (Odorizzi *et al.* 1998) using primers DN680p and DN693p, creating a PCR product with ends overlapping the ends of PvuII-cut vector pDN616. PCR template was eliminated by DpnI digestion. PvuII cuts on either side of the multiple cloning site (MCS), removing the MCS entirely, thus eliminating many redundant endonuclease cut sites. PCR product and PvuII-cut pDN616 were co-transformed into yeast to perform homologous recombination plasmid repair as described above. Transformants were selected on agar media lacking uracil to select circularized plasmids. pJM1 was modified to generate pJM3 by removal of the LoxP-flanked *CEN/ARSH4* locus by passaging pJM1 through Cre-expressing strain N2114Sm as described above, followed by transformation of TOP10F’ cells with N2114Sm-extracted plasmid in order to achieve abundant, monoclonal pJM3 candidates. To prepare pJM3 for chromosomal integration, pJM3 was linearized by SacII digestion of the unique cut site in the *PRC1* promoter sequence, generating linear ends capable of mediating homologous recombination at the *PRC1* chromosomal locus. Strains SEY6210 and MBY3 were both transformed using SacII-cut pJM3 using the high-efficiency protocol described above. Transformed yeast cells were selected on defined media lacking uracil and resulting colonies were re-struck to defined media lacking uracil to obtain monoclonal colonies derived from single cells. Correct integration of pJM3 at the *PRC1* locus was verified by “mapping” PCR of genomic DNA templates extracted from candidate colonies via the “smash and grab” protocol described above. Primer pairs DN4045p-DN4046p and DN4047p-DN4048p were used to confirm the correct integration of pJM3 on the upstream and downstream chromosomal sides of the *PRC1* locus, respectively.

Plasmids expressing the *L291A, L292A* mutant of *SEC17* (*sec17^LALA^*) driven by 500 bp native *SEC17 promoter* were constructed via an overlap extension PCR scheme in which the overlapping sequences of primers DN982p and DN983p introduced the *LALA* mutation. Flanking primers DN927p and DN928p included 35 and 34 bp, respectively, sequence overlapping the linear ends of SacI-digested pDN516, allowing insertion of *sec17^LALA^* PCR product into the pDN516 MCS via homologous recombination plasmid repair, resulting in pDN366. AatII digest of pDN366 linearized the plasmid and removed the *CEN/ARSH4* locus. Compatible AatII overhangs were ligated together using T4 ligase, omitting the *CEN/ARSH4* locus and resulting in pDN370. Insertion of 2 µ replication locus PCR product into AatII-digested pDN370 via Gibson cloning was performed essentially as described (Gibson *et al.* 2009), resulting in pDN369. PCR primers DN652p and DN653p used to amplify the 2 µ locus included 30 bp overhangs homologous to ends of AatII-digested pDN5xx vectors, yielding a PCR product compatible with either homologous recombination plasmid repair in yeast or *in vitro* overlap extension via Gibson cloning.

### Imaging

Pulse-chase labeling of log phase yeast with vacuolar fluorescent dye FM4-64 and fluorescence microscopy imaging were performed as described (Nickerson *et al.* 2012), except that cells were grown in nonselective YPD prior to labeling and imaging. DNA in agarose gels was stained using ethidium bromide and visualized using ultraviolet light in a BioRad Chemi-doc system with digital camera and Quantity One imaging software (BioRad, Hercules, CA, USA). All gel images were exported from Quantity One as .TIFF images, except Figure 2B, which was printed to photographic paper and later scanned in .TIFF format using an Epson flatbed scanner. Images were cropped using Adobe Photoshop CS6 (Adobe, San Jose, CA, USA). Fluorescence microscopy images were overlaid using ImageJ (NIH, https://imagej.nih.gov/ij/). Images were arranged as annotated figures using Canvas Draw 4 vector graphics software (Canvas GFX, Boston, MA, USA).

## Results and discussion

### pDN5xx & pDN6xx vector series

While several families of yeast shuttle vectors have been deployed over nearly four decades (Da Silva and Srikrishnan 2012), availability of the pRS series of vectors (Sikorski and Hieter 1989; Christianson *et al.* 1992) was a landmark in yeast genetics; the plasmids were rapidly adopted by the field and their use remains ubiquitous due to several useful features: i/yeast auxotrophic selection markers (*HIS3, TRP1, LEU2*, or *URA3*) are compatible with engineered auxotrophies in many of the most commonly used yeast laboratory strains (Sherman 2002); ii/plasmid selection in bacteria via ampicillin resistance; iii/bacterial origins of replication that produce high copy number and high plasmid yield upon extraction from bacteria; iv/a large polylinker or MCS with a selection of unique endonuclease cut sites to enable insertion of new DNA sequences; v/T3 and T7 phage promoters flanking the MCS to permit *in vitro* RNA transcription; and vi/a β-galactosidase coding region overlapping the MCS to permit colorimetric screening of bacterial colonies for successful integration of DNA insert.

Plasmids pRS403, pRS404, pRS405, and pRS406 were used as parent vectors in generating the pDN500- and pDN600-series. Actual DNA sequences of pRS family plasmids contain numerous sequence variations relative to the publicly available sequences (Chee and Haase 2012), most of which result in no apparent functional consequences. We independently examined sequences of our copies of pRS403, pRS404, pRS405, and pRS406 to generate accurate sequence files and maps (available in Supplementary Materials). Sequence variations were generally consistent with selected highlights previously reported (Chee and Haase 2012). Of note, while yeast selectable marker coding sequences for *HIS3* and *LEU2* contained many sequence variations from the original pRS maps, revised coding sequences were identical to reference genome sequences available at *Saccharomyces* Genome Database (SGD, www.yeastgenome.org). Coding sequences for *TRP1* and* URA3* were identical to those described in original pRS sequences.

**Figure 1:**
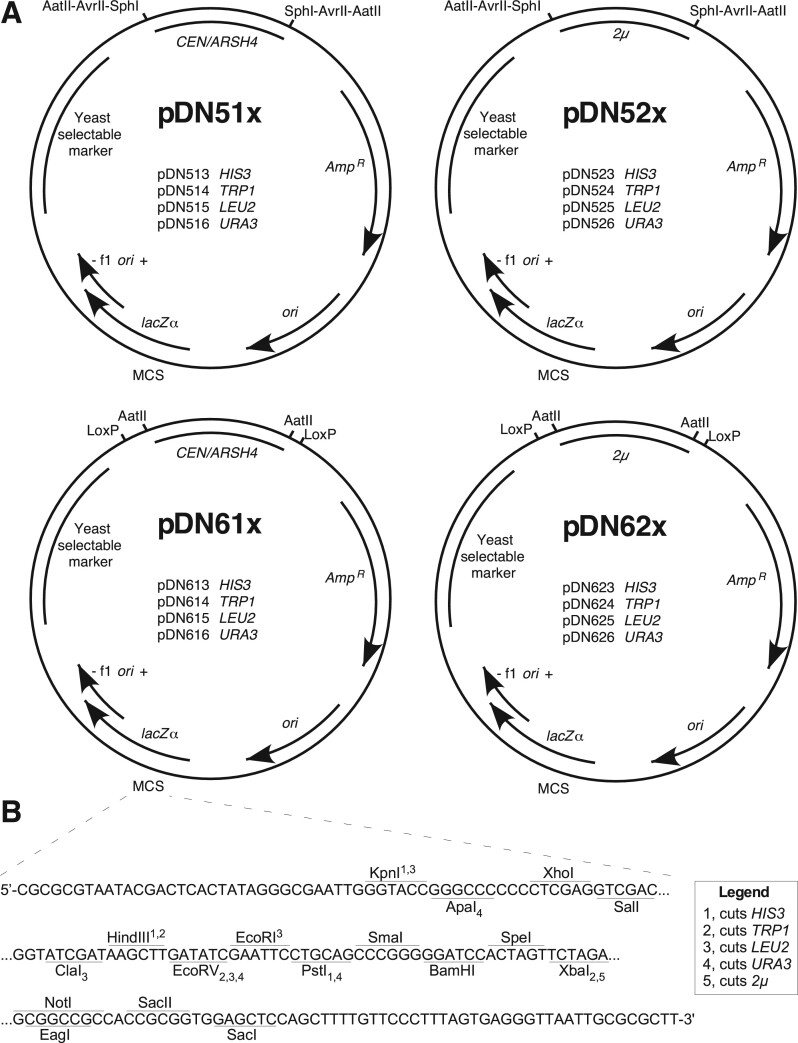
Functional maps for pDN5xx and pDN6xx series of low-copy and high-copy vectors. (A) Maps displaying consistent architectural features and specific functional differences of pDN5xx and pDN6xx families. Selected restriction enzyme cut sites and LoxP sequences flanking replication loci are displayed. (B) MCS in focus, displaying nucleotide sequence of single strand (template strand for *lacZα*, β-galactosidase) and unique restriction enzyme cut sites. Subscript and superscript numerals with each enzyme indicate capacity for enzyme to cut yeast selectable marker loci as indicated in legend. *CEN/ARSH4*, low copy yeast replication locus containing *CEN6* centromeric sequence and *ARSH4* ARS. 2 µ, yeast high copy replication locus including *STB* partitioning locus. *Amp^R^*, ampicillin resistance gene (β-lactamase). *ori*, high copy *E. coli* origin or replication. f1 *ori*, f1 bacteriophage origin of replication. Plasmid loci depicted at approximate scale. Full plasmid sequences and annotated maps are available in supplementary materials.

In designing the pDN500 and −600 series (Figure 1A and B), we honored the original numbering convention of the pRS series in which the second numeral indicates the yeast replication/segregation locus (“0” for none, “1” for centromeric, and “2” for 2 µ) and the third indicates the yeast selectable marker (“3” for *HIS3*, “4” for *TRP1*, “5” for *LEU2*, and “6” for *URA3*). In the pDN51x and pDN52x-series, yeast replication/segregation loci (hereafter referred to more simply as “replication loci”) are flanked by pairs of restriction endonuclease cut sites, enabling targeted *in vitro* removal of either *CEN* or 2 µ loci. The trio of AatII, AvrII, and SphI was selected because these enzymes are commonly used for laboratory cloning and lack cut sites elsewhere in the pRS and pDN family of vectors, excepting that AvrII cuts in the *HIS3* locus, consistent with characterization of the pRS vectors by (Chee and Haase 2012). Three pairs of flanking endonuclease cut sites were included to maximize the likelihood that at least one pair of cut sites should remain available to modify the replication locus in case further DNA inserts might contain cut sites for one or two of these enzymes. Importantly, removal of the replication locus by flanking restriction digest supports either elimination of the locus by plasmid re-circularization or substitution of an alternative replication locus.

The pDN61x and -62x series of vectors allow remodeling of yeast replication loci via a pair of parallel, flanking LoxP sequences, offering options to remodel the plasmid by Cre recombinase activity either *in vivo* or *in vitro.* The Cre-Lox recombination strategy offers the further advantage that the option to remodel a yeast replication locus will persist regardless of what further DNA sequences might be inserted into the plasmid, provided no additional LoxP sites are included. pDN600-series vectors also include a pair of flanking AatII cut sites on either side of the replication loci, so remodeling options available to the pDN500-series also apply.

It should be noted that the multiplicity of options (three different restriction enzymes or Cre recombinase treatment) for removing the replication loci from pDN51x, -52x, -61x, and 62x plasmids results in a collection of slightly different integrating plasmids that would all be accurately indicated by the pDN50x and pDN60x naming convention in which “0” indicates the absence of a replication locus. For example, while either Cre recombinase treatment or AatII digestion and recircularization of pDN614 would remove the *CEN/ARS4* locus to yield a pDN604 integrating vector, these would leave behind a single LoxP site or a reconstituted AatII cut site flanked by a pair of LoxP sites, respectively. Operators wishing to generate pDN60x vectors with maximum utility for future modification should follow the AatII digestion and recircularization steps previously mentioned; maximum utility in generating pDN50x vectors results from SphI digestion and recircularization.

### Conversion of replicating plasmids to integrating plasmids using Cre recombinase

**Figure 2:**
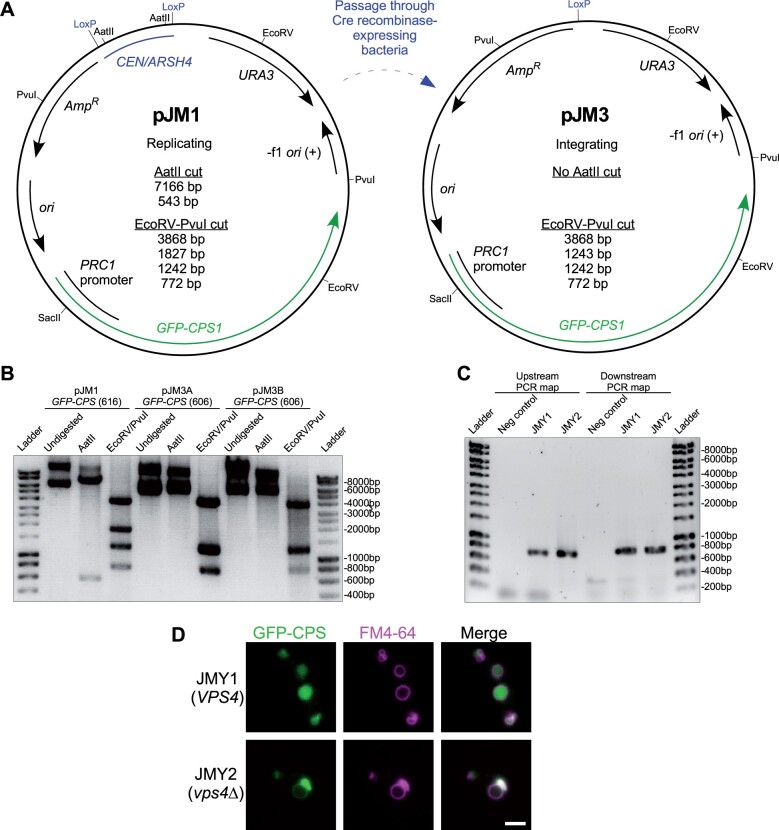
Conversion of replicating, episomal vector to integrating vector via Cre recombinase. (A) Plasmid maps of low copy replicating (pJM1) and integrating (pJM3) plasmids, including relevant endonuclease enzyme cut sites and predicted restriction fragment product sizes. Expression of *GFP-CPS1* is driven by the *PRC1* promoter. (B) Agarose gel electrophoretic analysis of restriction digest products derived from pre-Cre-treated plasmid pJM1 and post-Cre-treated plasmid pJM3 candidates A and B. Note that 1243 and 1242 bp fragments predicted from EcoRV-PvuI double digests of pJM3 appear as a single band. Undigested and uncut plasmids show high molecular weight bands representing supercoiled, nicked, and catenated circular DNA whose gel migration should not be compared to linear ladder size standards. Unlabeled DNA ladder bands are of length halfway between neighboring labeled bands. (C) Agarose gel electrophoretic analysis of “mapping” PCRs to confirm chromosomal integration of pJM3. pJM3 was digested with SacII to integrate at chromosomal *PRC1* promoter in strains SEY6210 and MBY3, generating strains JMY1 and JMY2 respectively. Chromosomal integrants were selected on media lacking uracil. Genomic DNA was extracted from candidate colonies to serve as PCR template. Upstream and downstream primer sets both employ a primer that anneals in the integrating plasmid and another that anneals in the neighboring chromosomal DNA, producing expected PCR products of 728 and 782 bp, respectively. Negative control samples used unmodified SEY6210 genomic DNA as template. (D) Fluorescence microscopy of FM 4-64-labeled, logarithmic phase yeast expressing chromosomally integrated pJM3. Cells were cultured in nonselective media prior to imaging. Scale bar = 1 µm.

We demonstrated working procedures for remodeling replication loci in the pDN600-series using plasmid pJM1 (Figure 2A), generated by incorporating a gene (*PRC1* promoter-driven *GFP-CPS*) encoding GFP-tagged transmembrane endosomal cargo reporter carboxypeptidase S into the centromeric, uracil-selected vector pDN616. Chromosomal integration is particularly useful for plasmids encoding fluorescent reporters, since uneven expression across a population of cells can result in a range of signal intensities and phenotypes (Da Silva and Srikrishnan 2012; Chou *et al.* 2015; Lee *et al.* 2015). We converted pJM1 into a replication-deficient, integrating plasmid (pJM3) via Cre recombinase activity toward the LoxP-flanked *CEN/ARSH4* locus. We passaged pJM1 through *E. coli* strain N2114Sm (Seifert *et al.* 1986) that stably expresses Cre. Plasmid DNA was extracted from ampicillin-selected N2114Sm colonies and used to transform *E. coli* strain TOP10F’ for the dual purpose of filtering the polyclonal plasmid population immediately derived from N2114Sm and achieving a higher yield of plasmid DNA. Restriction analysis of the resulting pJM3 candidates revealed that neither candidate retained the AatII cut sites that flank the *CEN/ARSH4* locus in pJM1 (Figure 2B). Digestion with a combination of EcoRV and PvuI further confirmed that a pJM1 restriction product containing the *CEN/ARSH4* locus (1834 bp) was absent in pJM3 candidates, but that the restriction fragment at ∼1250 bp in pJM3 had doubled in relative intensity, indicating the presence of two fragments, consistent with the 1827 bp fragment being reduced to 1243 bp by removal of the *CEN/ARSH4* sequence by successful Cre-Lox recombination.

We confirmed competence of pJM3 as an integrating plasmid by cutting at the unique SacII recognition site in the *PRC1* promoter sequence driving expression of *GFP-CPS*, producing linearized pJM3 with end sequences to direct integration via homologous recombination into the chromosomal *PRC1* promoter. We transformed linearized pJM3 into wild-type yeast and a *vps4Δ* mutant strain that suffers an endosome maturation defect preventing formation of luminal vesicles (Babst *et al.* 1997), selecting transformed cells on media lacking uracil and extracting genomic DNA from resulting candidate colonies for analysis. Correct integration of pJM3 at the *PRC1* promoter locus was confirmed via “mapping” PCR (Figure 2C) that confirmed correct orientation of the integration cassette relative to flanking chromosomal DNA on either side, “upstream” facing chromosomal *PRC1* promoter sequence and “downstream” facing chromosomal *PRC1* coding sequence. Correct orientation of primer pairs (one annealing in chromosomal sequence, the other to integrating plasmid sequence) resulted in upstream and downstream mapping PCR products of the predicted ∼800 bp size in both wild type and *vps4Δ* mutant cells transformed with SacII-cut pJM3, confirming new yeast strains JMY1 and JMY2, respectively. Cells expressing GFP-CPS were cultured in complex, nonselective media prior to pulse-labeling with a fluorescent endocytic membrane dye, FM4-64 (Vida and Emr 1995) to stain the perimeter of the yeast vacuole. GFP-CPS transits via the biosynthetic pathway from Golgi to endosome where it is sorted into luminal endosomal vesicles (Odorizzi *et al.* 1998). Luminal vesicles and GFP-CPS cargo are delivered to the vacuole lumen when endosomes fuse with the vacuole, so in wild-type cells GFP signal appeared inside the FM4-64-stained vacuole membrane (Figure 2C). Loss of the gene *VPS4* (*vps4Δ*) disrupts the ability of endosomes to invaginate and form luminal vesicles, so GFP-CPS remains at the outer membrane of endosomes and is delivered instead to the outer membrane of the vacuole, co-localizing with FM4-64 at the vacuole membrane and at perivacuolar endosomal compartments (Figure 2C). These observations are consistent with previous studies (Odorizzi *et al.* 1998) and confirm performance of the pDN600 series in converting to integration-competent vectors.

**Figure 3:**
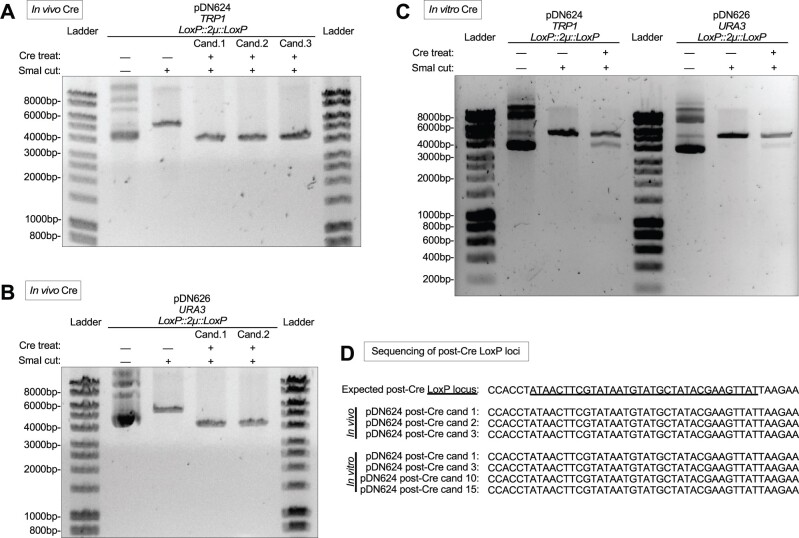
Cre-mediated removal of 2 µ replication locus *in vivo* and *in vitro*. (A,B) Agarose gel electrophoretic confirmation of removal of 2 µ replication locus after passage of pDN624 (A) or pDN626 (B) through Cre-expressing bacterial strain N2114Sm. Each candidate represents a unique plasmid isolated from a single N2114Sm colony and subsequently transformed into and re-isolated from TOP10F’ cells. SmaI-cut (linearized) pDN624 produces predicted bands of 5693 bp and 4303 bp before and after removal of 2 µ locus, respectively. SmaI-cut (linearized) pDN626 produces predicted bands of 5792 bp and 4402 bp before and after removal of 2 µ locus, respectively. (C) Agarose gel electrophoretic confirmation of removal of 2 µ replication loci after *in vitro* treatment of pDN624 and pDN626 with Cre recombinase. Cre-treated samples represent polyclonal populations that include both unmodified (5693 bp for pDN624; 5792 bp for pDN626) and modified plasmids (4303 bp for pDN624; 4402 bp for pDN626). (D) Sanger DNA sequencing of Cre-treated pDN624 plasmid candidates in panels A and C confirming absence of 2 µ locus and remainder of a single LoxP site. Polyclonal *in vitro* Cre-treated pDN624 sample was transformed into TOP10F’ cells and plasmid candidates were purified from single colonies. Fifteen (15) *in vitro* candidates were pre-screened by restriction analysis to confirm linearized plasmid length consistent with removal of 2 µ locus, yielding four (4) candidates for sequencing. Unlabeled DNA ladder bands are of length halfway between neighboring labeled bands.

We further examined whether Cre would remove LoxP-flanked 2 µ replication loci. We passaged the high copy vectors pDN624 (Figure 3A) and pDN626 (Figure 3B) through Cre-expressing bacteria via the same procedure described above. Restriction analysis using endonuclease SmaI revealed that all Cre-treated plasmid candidates had been reduced in length ∼1400 bp compared to untreated plasmids, consistent with the predicted loss of 1390 bp due to Cre recombination of the *LoxP::2*µ*::LoxP* cassette. Purified Cre enzyme is readily available from commercial suppliers, so we also examined whether LoxP-flanked yeast replication loci could be removed *in vitro*. Cre-treatment of high copy plasmids pDN624 and pDN626 for only 30 min resulted in modification of a substantial subpopulation of the plasmids (Figure 3C). Treated samples possessed bands representing unmodified pDN624 and pDN626 as well as an additional band ∼1400 bp shorter in length, consistent with the predicted loss of 1390 bp from each after *LoxP::2*µ*::LoxP* recombination. Sanger DNA sequencing of both *in vivo and in vitro* Cre-treated pDN624 plasmid candidates (Figure 3D) confirmed that the observed 1390 bp reduction of candidate plasmid lengths correlated with loss of the 2 µ locus and retention of a single *LoxP* site, as would be expected after Cre-mediated removal of a LoxP-flanked locus. Users of the pDN600 series therefore have the option to conduct Cre-mediated remodeling of the yeast replication locus either *in vivo* or *in vitro*.

### Generation of integrating plasmids by restriction digest to excise plasmid replication loci

**Figure 4:**
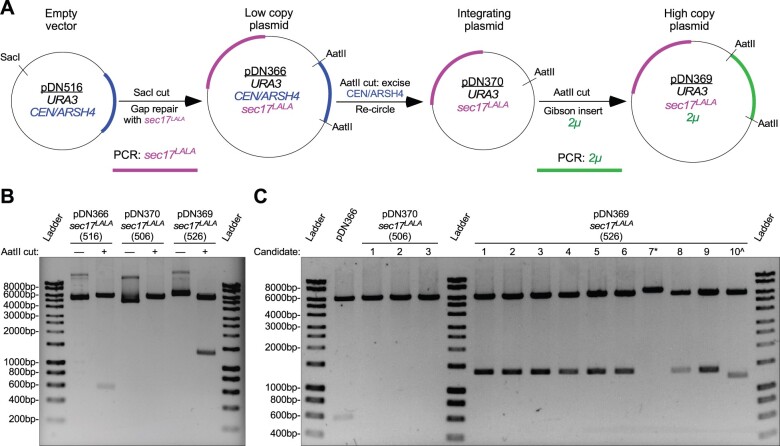
Example workflow to generate low copy, high copy, and integrating plasmids from a common precursor. (A) Workflow schematic representing modification of original low copy replicating vector by insertion of a PCR product at MCS containing SacI cut site, followed by removal of original replication locus and replacement with high copy replication locus. (B) Agarose gel electrophoresis and restriction enzyme analysis of plasmids resulting from demonstrated workflow. Observed AatII restriction fragments conform to predicted sizes: 6174 bp and 563 bp for pDN366; 6174 bp for pDN370; and 6174 bp and 1369 bp for pDN369. (C) Agarose gel electrophoretic analysis of efficiency of removal of *CEN/ARSH4* replication locus and replacement with 2 µ replication locus. All samples shown were digested with AatII to linearize vector (no replication locus) or cut on either side of replication locus. * and ^ symbols indicate failed pDN369 candidates. All plasmid samples presented for analysis are monoclonal plasmid populations purified from transformed bacteria grown from single cell colony isolates. Unlabeled DNA ladder bands are of length halfway between neighboring labeled bands.

We explored the plasticity of the pDN500-series by generating a full suite of replicating and integrating plasmid vectors expressing a mutant allele (*L291A, L292A—or “LALA”*) of the SNARE disassembly adaptor protein Sec17 (Schwartz *et al.* 2017). We generated a *sec17^LALA^* PCR product with ends homologous to the ends of SacI-digested pDN516, inserting *sec17^LALA^* into pDN516 via co-transformation into yeast cells for plasmid gap repair by homologous recombination (Figure 4A). The resulting low copy, centromeric plasmid (pDN366) was subsequently converted to a nonreplicating, integrating plasmid by digesting pDN366 with AatII and re-circularizing with T4 ligase to remove the *CEN/ARSH4* locus, resulting in pDN370. In order to generate a high copy plasmid to overexpress *sec17^LALA^*, pDN370 was again cut with AatII to make linear ends available for insertion of a PCR-generated 2 µ locus via Gibson assembly (Gibson *et al.* 2009) to yield pDN369. Operators should note that Gibson assembly and homologous recombination cloning in yeast cells both rely upon overlapping homologous sequences at the ends of vector and insert, so vector and insert for pDN369 could have been assembled using either approach.

All three versions of the *sec17^LALA^* plasmid suite produce a 6185 bp band upon AatII digestion (Figure 4B), representing the *sec17^LALA^* gene insert and the remainder of the common plasmid backbone that lacks the yeast replication locus. pDN366 and pDN369 also produce AatII restriction fragments at 563 or 1369 bp, representing *CEN/ARSH4* or 2 µ loci, respectively. Candidate plasmids examined after recircularization of pDN366 to produce pDN370 all lack the 563 bp *CEN/ARSH4* locus (Figure 4C), which was expected given the robustness of the recircularization technique. Screening 10 candidate plasmids for high copy pDN369 generated by Gibson assembly also revealed a high degree of successful 2 µ locus insertion (80%).

### Considerations and alternative approaches

The workflow described in Figure 4 works equivalently if the starting vector is high copy (2 µ) instead of low copy (*CEN/ARSH4*); such an alternative workflow was used to generate LUCID (Luciferase Reporter of Intraluminal Deposition) family cargo transport reporter plasmids (Nickerson *et al.* 2012; Nickerson and Merz 2015) in which multiple genes expressing chimeric reporter enzymes were inserted at the MCS, so the ability to remodel the replication loci of the plasmids was preferable to subcloning large, multi-component gene inserts.

A further convenience of a standard family of yeast shuttle vectors with ability to remodel the replication loci is the limited number of needed reagents for a research lab to stock. Indeed, remodeling operations demonstrated in this study could be performed using the enzymes AatII, Cre, and T4 ligase, plus frozen stocks of *CEN/ARSH4* and 2 µ PCR products ready to insert.

Alternative approaches exist to modify the original pRS series of shuttle vectors to change their copy number or replication competency, even without the deliberately engineered mechanisms incorporated into the pDN series. For example, the dual cutter restriction enzymes PvuI and PvuII both have cut sites that flank the pRS and pDN series MCS at a distance of 100 bp or more, creating a restriction fragment containing the gene insert that can recombine (*e.g.*, via homologous recombination) with other pRS or pDN family vectors linearized by cutting in the MCS. Unique restriction sites in or near the *CEN/ARS4* loci in the original pRS41x series can also be used to remove the *CEN/ARS4* locus and linearize the vector for recircularization or insertion of a high copy locus. For example, unique cut sites for SwaI (found in the *CEN6* locus) and PfoI (found between the replication locus and yeast selectable marker) are available in each of the pRS41x, pDN51x, and pDN61x series plasmids, and such a double digest would remove essentially all of the *CEN/ARS4* locus. However, a recurring theme in these described alternative approaches is dependency on restriction sites that often lose their unique or dual status upon introduction of gene(s) of interest into the shuttle vector. Availability in the pDN500 or pDN600 series of multiple, functionally redundant restriction sites or LoxP sites flanking the replication loci ensures that operators should retain a convenient, standardized option to remodel the replication locus regardless of what other sequences might have been inserted.

A practical consideration in remodeling the replication and partitioning loci of yeast plasmids is that operators are safest avoiding simultaneous presence of both *CEN/ARS* and 2 µ loci in the same plasmid. For example, we would not recommend attempting to convert a low copy plasmid to high copy by inserting a 2 µ locus without deleting all or most of the *CEN* locus. The *CEN* locus functions both in mitotic/meiotic partitioning and in constraining plasmid copy number. *CEN*-*2*µ composite plasmids were found (Tschumper and Carbon 1983) to be maintained at low copy number, so adding a 2 µ replication locus to a *CEN* plasmid would not necessarily render it high copy. *CEN*-*2*µ composite plasmids were further found to present risk of genetic instability (Tschumper and Carbon 1983). Even in such a circumstance where plasmid-borne centromeric loci could be present at moderately high copy numbers, these would be expected to result in both plasmid and chromosomal genetic instability (Futcher and Carbon 1986). Such risks reinforce the utility in the pDN vector series of providing reliable, redundant mechanisms to remove the entirety of the low copy *CEN/ARS4* or high copy 2 µ loci before inserting an alternative replication locus.

Future improvements to shuttle vector systems could merge the benefits of targeted removal or remodeling of both the yeast replication loci and the yeast selectable markers. The use of Cre-Lox recombination in the pDN600-series presents an incompatibility with the commonly used Cre-Lox removal of selectable marker loci, but there are several alternative recombinase enzymes and recognition sequences available to incorporate.

## Data availability

Strains and plasmids are available upon request. Supplementary materials include oligonucleotide/primer sequences and descriptions (Supplementary Table S1) and vector sequences and maps for the pRS40x, pDN51x, pDN52x, pDN61x, and pDN62x vector series in SnapGene file format (.dna). Plasmid vectors in the pDN51x, pDN52x, pDN61x, and pDN62x vector series will be deposited at Addgene (addgene.org, Cambridge, MA, USA). Direct reagent requests to the Nickerson Lab should please provide a self-addressed, stamped envelope, mailed to “Attn: Nickerson, 5500 University Pkwy, CSUSB Biology Dept, Rm BI-302, San Bernardino, CA 92407-2318.” Some plasmids in the pDN61x and pDN52x series were first reported in earlier studies (referenced in Table 1); users of these resources may reference either this study (preferred) or the original report.

Supplementary materials available at *G3* online. 

## Author contributions

D.P.N. conceived of the project. D.P.N. and M.A.Q. conceived and designed experiments. D.P.N., J.M.M. and M.A.Q. performed experiments and analyzed results. D.P.N. wrote the manuscript with editing contributions from M.A.Q. and J.M.M.

## Funding

This work was supported by grants from the CSUSB Office of Student Research to M.A.Q. and D.P.N., charitable donations to the Nickerson Lab Research Fund (CSUSB), NIH/NIGMS (National Institutes of Health/National Institute of General Medical Sciences) RO1 GM077349 to A.J.M. (University of Washington), and NIH/NIGMS SC2 GM140979 to D.P.N. Content is solely responsibility of the authors and does not necessarily represent official views of the National Institutes of Health.

## Conflicts of interest

The authors declare that there is no conflict of interest.

## Supplementary Material

jkab336_Supplementary_Files

jkab336_Supplementary_Table
