# Supplementary material for: Rapid conversion of replicating and integrating Saccharomyces cerevisiae plasmid vectors via Cre recombinase
Source: G3 (Bethesda). 2021 Oct 2;11(12):jkab336. doi: 10.1093/g3journal/jkab336 (PMC8664424; doi:10.1093/g3journal/jkab336)
Supplement: jkab336_Supplementary_Table [file jkab336_supplementary_table.docx]

**SUPPLEMENTAL TABLE – Nickerson et al. 2021**

**Table S1**. **Primer sequences used in this study.**

DN648p: Rev, PCR amplify *CEN/ARSH4* sequence, incorporate LoxP sequence and AatII restriction site, mediate repair of AatII-digested pRS40x vector.

5’**GGTTAATGTCATGATAATAATGGTTTCTTA***ataacttcgtatagcatacattatacgaagttat*GACGTCggacggatcgcttgcctg-3’

DN649p: Fwd, PCR amplify *CEN/ARSH4* sequence, incorporate LoxP sequence and AatII restriction site, mediate repair of AatII-digested pRS40x vector.

5’**CGCGCACATTTCCCCGAAAAGTGCCACCT***ataacttcgtataatgtatgctatacgaagttatt*GACGTCcccgaaaagtgccacctg-3’

DN652p: Fwd, PCR amplify *CEN/ARSH4* sequence, incorporate triplicate restriction sites, mediate repair of AatII-digested pRS40x or pDN50x vectors.

5’**CACATTTCCCCGAAAAGTGCCACCTGACGT**CCTAGGCATGCggtccttttcatcacgtgc-3’

DN653p: Rev, PCR amplify *CEN/ARSH4* sequence, incorporate triplicate restriction sites, mediate repair of AatII-digested pRS40x or pDN50x vectors.

5’**ATGTCATGATAATAATGGTTTCTTAGACGT**CCTAGGCATGCgataataatggtttcttag-3’

DN1016p: Fwd, PCR amplify *2µ* sequence, incorporate triplicate restriction sites, mediate repair of AatII-digested pRS40x or pDN50x vectors.

5’**CACATTTCCCCGAAAAGTGCCACCTGACGT**CCTAGGCATGCaacgaagcatctgtgcttcatt-3’

DN1017p: Rev, PCR amplify *2µ* sequence, incorporate triplicate restriction sites, mediate repair of AatII-digested pRS40x or pDN50x vectors.

5’**ATGTCATGATAATAATGGTTTCTTAGACGT**CCTAGGCATGCgatccaatatcaaaggaaatg-3’

MQ1p: Fwd, PCR amplify *2µ* locus, mediate insertion into AatII-digested pDN61x family vectors to create pDN62x family vectors. 5’**ATAATGTATGCTATACGAAGTTATTGACGT**caacgaagcatctgtgcttcattttg-3’

MQ2p: Rev, PCR amplify *2µ* locus, mediate insertion into AatII-digested pDN61x family vectors to create pDN62x family vectors.

5’**TATAGCATACATTATACGAAGTTATGACGT**cgatccaatatcaaaggaaatgatagc-3’

DN661p: Sequencing primer for yeast replication loci, anneals near yeast selectable marker locus.

5’tacaatctgctctgatgcc-3’

DN837p: Sequencing primer for yeast replication loci, anneals in *AmpR* promoter.

5’ttattgaagcatttatcaggg-3’

DN680p: Rev, PCR amplify 200 bases of *CPS1* terminator to copy *PRCpr::GFP-CPS1* cassette, mediate repair of PvuII-digested pDN616.

5’**gatcggtgcgggcctcttcgctattacgccag**taaattttgatttgacacttg-3’

DN693p: Fwd, PCR amplify 455 bases of *PRC1* promoter to copy *PRC1pr::GFP-CPS1* cassette, mediate repair of PvuII-digested pDN616.

5’**cctctccccgcgcgttggccgattcattaatgcag**attgacagagcagtatgtgagg-3’

DN927p: Fwd, PCR amplify 500 bp *SEC17* promoter, mediate gap repair into SacI-digested pDN516.

5’**CTAGTTCTAGAGCGGCCGCCACCGCGGTGGAGCTC**ttctttgtcaattgcatctcta-3’

DN928p: Rev, PCR amplify 300 bp of *SEC17* terminator, mediate gap repair into SacI-digested pDN5xx.

5’**TAACCCTCACTAAAGGGAACAAAAGCTGGAGCTC**ggaagatccttacattacacg-3’

DN982p: Fwd, introduce *L291A L292A* (‘LALA’) mutation into *SEC17* via sequence overlap extension PCR, overlaps with DN983p.

5’ATCCAGCAACAAGAAGATGAT GCG GCA TGA acggcatatacttacgcgca-3’

DN983p: Rev, introduce *L291A L292A* (‘LALA’) mutation into *SEC17* via sequence overlap extension PCR, overlaps with DN982p.

5’TGCGCGTAAGTATATGCCGT TCA TGC CGC atcatcttcttgttgctggat-3’

DN4045p: Fwd, upstream mapping PCR to confirm pJM3 integration at chromosomal *PRC1* locus, anneals in chromosomal *PRC1* promoter

5’gaaggcattggttagggtctagta-3’

DN4046p: Rev, upstream map PCR to confirm pJM3 integration at chromosomal *PRC1* locus, anneals in plasmid *GFP* coding sequence.

5’ccttcaccctctccactgacag-3’

DN4047p: Fwd, downstream map PCR to confirm pJM3 integration at chromosomal *PRC1* locus, anneals in plasmid *ori* locus*.*

5’caggggggcggagcctatgg-3’

DN4048p: Rev, downstream map PCR to confirm pJM3 integration at chromosomal *PRC1* locus, anneals in chromosomal *PRC1* coding sequence.

5’gtgtagtggacaggcctagtcc-3’

DN4086p: Sequencing, anneals upstream of selectable marker loci in pRS / pDN vector series.

5’acagcttgtctgtaagcggatg-3’

DN4087p: Sequencing, anneals Rev in *AmpR* coding sequence.

5’ggcaaaatgccgcaaaaaaggg-3’

DN4088p: Sequencing, anneals Rev in *AmpR* coding sequence.

5’gttaatagtttgcgcaacgttgt-3’

DN4089p: Sequencing, anneals Rev in *ori* locus.

5’tgcaagcagcagattacgcgc-3’

DN4090p: Sequencing, anneals Rev in *ori* locus.

5’catcacaaaaatcgacgctcaag-3’

DN4091p: Sequencing, anneals Rev in *LacZ* coding sequence near to MCS.

5’cttttgttccctttagtgaggg-3’

DN4092p: Sequencing, anneals Rev in *f1 ori*.

5’ctcattttttaaccaataggccg-3’

DN4093p: Sequencing, anneals Fwd in *f1 ori*.

5’gtttttcgccctttgacgttgg-3’

DN4094p: Sequencing, anneals Fwd in *HIS3* promoter.

5’ggcaagataaacgaaggcaaag-3’

DN4095p: Sequencing, anneals Fwd in *HIS3* coding sequence.

5’tttcgaacaggccgtacgca-3’

DN4096p: Sequencing, anneals Fwd in *TRP1* coding sequence.

5’TTTCACAGGTAGTTCTGGTCCA-3’

DN4097p: Sequencing, anneals Fwd in *TRP1* coding sequence.

5’actcgtatttccaaaagactgca-3’

DN4098p: Sequencing, anneals Rev in *LEU2* terminator.

5’atttgcttacctgtattcctttac-3’

DN4099p: Sequencing, anneals Rev in *LEU2* coding sequence.

5’gtacaaaccaaatgcggtgttc-3’

DN4100p: Sequencing, anneals Rev in *LEU2* coding sequence.

5’gcaaattgtggcttgattggag-3’

DN4101p: Sequencing, anneals Rev in *LEU2* promoter.

5’gctgaaatgtaaaaggtaagaaaag-3’

DN4102p: Sequencing, anneals Fwd in *URA3* coding sequence.

5’taatatcatgcacgaaaagcaaac-3’

DN4103p: Sequencing, anneals Fwd in *URA3* coding sequence.

5’gcagaattgtcatgcaagggc-3’

Sequences to mediate homologous recombination shown in **bold**. LoxP sequences shown in *italics*. Restriction site sequences shown as underlined. PCR annealing sequences shown in lowercase.
